# Artificial intelligence for prediction of biological activities and generation of molecular hits using stereochemical information

**DOI:** 10.1007/s10822-023-00539-9

**Published:** 2023-10-17

**Authors:** Tiago O. Pereira, Maryam Abbasi, Rita I. Oliveira, Romina A. Guedes, Jorge A. R. Salvador, Joel P. Arrais

**Affiliations:** 1 Centre for Informatics and Systems, Department of Informatics Engineering, University of Coimbra, Coimbra, Portugal; 2 Applied Research Institute, Polytechnic Institute of Coimbra, Coimbra, Portugal; 3 Laboratory of Pharmaceutical Chemistry, Faculty of Pharmacy, University of Coimbra, Coimbra, Portugal; 4 Center for Neuroscience and Cell Biology, Center for Innovative Biomedicine and Biotechnology, Coimbra, Portugal; 5 Research Centre for Natural Resources Environment and Society (CERNAS), Polytechnic Institute of Coimbra, Coimbra, Portugal

**Keywords:** Deep reinforcement learning, De novo drug design, Attention mechanism, Stereochemical information, Interpretability

## Abstract

In this work, we develop a method for generating targeted hit compounds by applying deep reinforcement learning and attention mechanisms to predict binding affinity against a biological target while considering stereochemical information. The novelty of this work is a deep model Predictor that can establish the relationship between chemical structures and their corresponding $pIC_{50}$ values. We thoroughly study the effect of different molecular descriptors such as ECFP4, ECFP6, SMILES and RDKFingerprint. Also, we demonstrated the importance of attention mechanisms to capture long-range dependencies in molecular sequences. Due to the importance of stereochemical information for the binding mechanism, this information was employed both in the prediction and generation processes. To identify the most promising hits, we apply the self-adaptive multi-objective optimization strategy. Moreover, to ensure the existence of stereochemical information, we consider all the possible enumerated stereoisomers to provide the most appropriate 3D structures. We evaluated this approach against the Ubiquitin-Specific Protease 7 (USP7) by generating putative inhibitors for this target. The predictor with SMILES notations as descriptor plus bidirectional recurrent neural network using attention mechanism has the best performance. Additionally, our methodology identify the regions of the generated molecules that are important for the interaction with the receptor’s active site. Also, the obtained results demonstrate that it is possible to discover synthesizable molecules with high biological affinity for the target, containing the indication of their optimal stereochemical conformation.

**Supplementary Information:**

The online version contains supplementary material available at 10.1007/s10822-023-00539-9.

## Introduction

The development of effective and safe candidate drugs is becoming an increasingly complex, time-consuming and expensive process. On average, it takes 12 years and $2.6 billion to bring a new drug into the market [1]. Despite the breakthroughs in computational methods and a better understanding of biological processes related to the onset of diseases, the approval rate of candidate drugs by regulatory authorities remains low. When researching therapeutic responses to cancer, the paradigm is no different. Cancer is still a major concern worldwide, as demonstrated by the high incidence and mortality rates [2]. As a result, it is essential to devise effective computational methods to promote the efficient discovery of safe therapeutical solutions for cancer treatment.

Obtaining a limited but promising initial set of hit compounds is a significant aspect that can contribute to the efficiency of the drug development process. In this regard, it is necessary to find molecules that exhibit a strong affinity for the target and that have physicochemical properties ensuring their bioavailability while minimizing toxicity to the organism [3]. As a result of this task’s complexity, the selection of drug precursors is predominantly based on trial and error, expert intuition, and serendipity-based methods [4]. Therefore, by combining domain expertise with data-driven methods, computational methods can play a pivotal role in the early stages of the drug development process, specifically through the focused identification of promising compounds.

One of the most widely adopted computational approaches is the virtual high-throughput screening, which allows the identification of interesting compounds from the analysis of vast libraries of known compounds [5]. Recently, this procedure has been enhanced with the advent of computer-aided drug design (CADD) applying deep learning [6], tools such as molecular docking [7], and molecular dynamic simulations [8]. Nevertheless, its efficiency and usefulness remain limited to a few specific contexts due to the vastness of the drug-like chemical space. The alternative approach is the de novo generation of chemical structures. In this case, CADD strategies explore the chemical space freely to generate new compounds from scratch. There are several approaches for implementing de novo generation, such as inverse quantitative structure-activity relationship (QSAR) [9], evolutionary algorithms [10], or generative models based on machine learning and deep learning architectures [11, 12].

Machine-learning-based methods have become popular in this field due to the greater availability of cheminformatic data and to the development of models adept at extracting hidden patterns from these raw data [13]. There are different ways to represent molecules computationally, but the most used are the simplified molecular-input line-entry system (SMILES) and graph notation. SMILES notation encodes the molecule into a sequence of characters, while graphs store the atom, bond type, and connectivity information of a molecule in multidimensional arrays [14]. Regarding the data-driven methods, we can highlight the generative models based on deep learning. Generative models are applied in hit compound discovery due to their ability to learn the distributions embedded in training data, which then allows the generation of similar novel instances with bespoken molecular properties. An illustrative example is the generative adversarial network (GAN), whose generative component seeks to learn how to generate new molecules that are similar to the given molecular set [15]. This set contains molecules with optimized properties, and the generator tries to create compounds that mimic the same distribution while the discriminator attempts to determine the origin of the sampled compounds [16, 17]. Variational autoencoders (VAE) have been used to generate optimized hits by bidirectionally mapping molecules to latent subspaces where it is easier to improve their drug-likeness, and physicochemical properties [18]. Another widely explored approach is the recurrent neural networks (RNNs) because of their capacity to learn the syntax for constructing valid molecules using SMILES notation [14]. Moreover, RNN-based models are easily integrable with different optimization strategies, such as reinforcement learning (RL), to produce compounds with optimized properties [12, 19].

These methodologies were able to deliver effective results when generating compounds with individual properties optimized, especially considering physicochemical objectives. Nevertheless, most approaches fall short in critical aspects for the successful progression of the drug development process. Firstly, candidate drugs must satisfy a set of fundamental characteristics to become viable drugs. For instance, to be considered a promising hit, a molecule must be synthesizable in the laboratory and have a significant biological affinity for the target. Therefore, the generation of candidate hits has an inherently multi-objective nature that is often neglected. Other common problems in the output of molecular generative frameworks are the absence of stereochemical information and the lack of explanation on how the drug-target interaction will possibly occur. Stereochemistry encodes important information for the biological activity of molecules, and, as a result, it must be considered when designing small molecules to interact with targets. The lack of explicability provided by the computational methods is noticeable as it is not possible to specify which regions of the molecule will be involved in binding. This information would be valuable in the compound’s further optimization steps as well as in validating the proposed model’s quality.

Herein, we propose a generative framework of compounds with optimized properties by applying prominent deep-learning methodologies such as RNNs and attention mechanisms. In short, the RNN-based generator model will explore the chemical space through RL, guided by a drug-target biological affinity predictor incorporating an attention layer. Also, the novelty of this approach is the implemented self-adaptive multi-objective optimization strategy, the incorporation of the spatial arrangement of atoms around the identified hit molecules and the indication of the most important zones for interaction with the target using the attention mechanism. The practical case explored was the generation of putative hit compounds that can inhibit the USP7 target due to the importance of this target for the proliferation of different types of tumours [20]. USP7 is a cysteine protease whose catalytic domain conformation shows significant structural flexibility upon substrate binding. The deubiquitination catalyzed by USP7 is a multi-step process in which cysteine 223 (Cys223) is at the core of a nucleophilic attack [21]. More precisely, through the establishment of a hydrogen bond between aspartic acid 481 (Asp481) and histidine 464 (His464), Asp481 will assist the orientation of the imidazole ring of His464 into a favourable location toward Cys223. This results in the deprotonation of the thiol of Cys223, revealing a nucleophile that will perform a nucleophilic attack toward the carbonyl group of the isopeptide bond between the substrate N-terminal lysine and the ubiquitin C-terminal glycine to give rise to a tetrahedral intermediate with ubiquitin. Lastly, Cys223 and ubiquitin are released through hydrolysis of the thioester bond between USP7 and ubiquitin C-terminal glycine [21, 22].

The main objective of this work is to employ the state-of-the-art de novo molecular generation methodologies and narrow the most significant gaps related to the synthesizability of compounds, stereochemical organization and interpretability of the results.

## Materials and methods

**Fig. 1:**
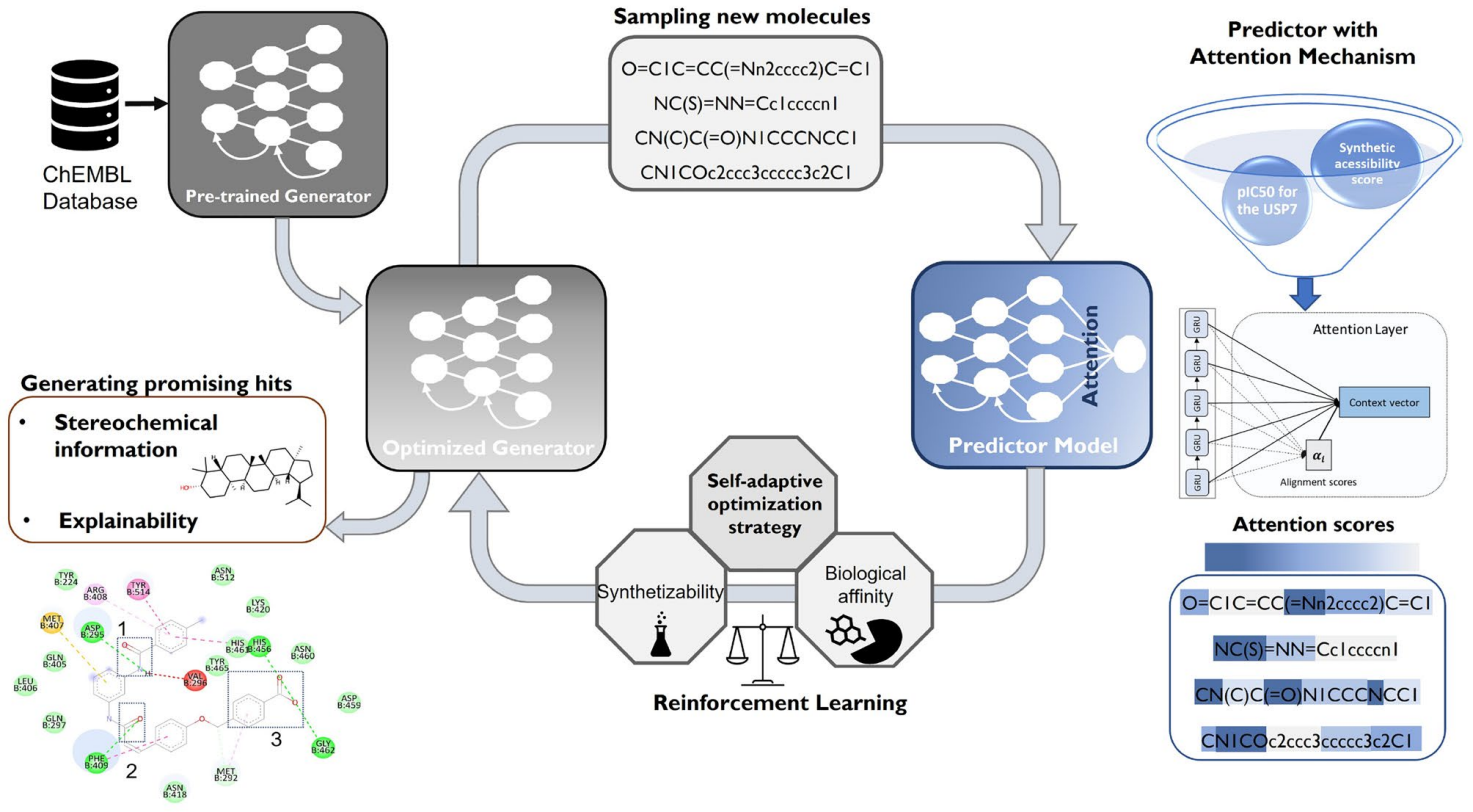
General workflow. Implementation steps of the model for generating molecules with desired properties, stereochemical information, and indication of the key active sites

The proposed framework consists of a Generator of molecular structures and a Predictor, which estimates the biological activity of the compounds against the USP7 target. As indicated in Fig. 1, the two DL models are combined to implement a conditioned generation dynamic through RL. The objective is for the Generator to learn how to explore the most promising zones of the chemical space while considering the properties to be optimized. At the end of the process, it will be possible to identify the spatial configuration of the atoms and the most important interaction regions for the best hits generated. Nevertheless, before integrating the RL dynamics, the Generator and the Predictor will be pre-trained. The former is trained to generate chemically valid compounds, i.e., it will acquire knowledge about the vast chemical space, whereas the latter will learn to predict the binding affinity between the sampled molecules and the USP7 target. At the end of the optimization process, we performed docking experiments using the best compounds, i.e., the ones with higher biological affinity against the USP7, to simulate the potential interactions and validate the entire framework.

### Pretraining of the generator

#### Dataset

The dataset of chemical information used to train the Generator was obtained from the ChEMBL database. We assembled 1,179,477 SMILES from the ChEMBL22 version. To ensure that the initial chemical space was within the drug-like region, the selected small molecules had a molecular weight ranging from 200 to 600 g/mol and a partition coefficient (logP) ranging from − 2 to 6. Additionally, the input dataset was filtered using the RDKit library, to exclude the molecules that could not be parsed and canonicalized by this tool.

#### The generator architecture

The Generator is built with recurrent architectures due to their ability to operate with sequential inputs, as is the case of the SMILES notation [23]. The pre-processing steps required for this input include tokenization and padding of each sequence. The training compounds are then encoded using an embedding layer and processed by a set of two long short-term memory (LSTM) layers. After the extraction of the main features by these layers, the next token of the sequence is predicted based on the output of a softmax-activated dense layer. Hence, the sequence analysis is performed token-by-token, which means that the model should learn how to maximize the probability of the true token given the preceding context. The optimal implementation parameters of the Generator are indicated in Table S1 of the Supporting Information. As shown in Eq. 1, the loss function is the categorical cross-entropy since we intend to minimize the distance between the distribution predicted by the model ($\hat{y}_{t}$) and the true output ($y_{t}$).

1 $$\begin{aligned} J(\theta )=-\frac{1}{T} \sum _{t=1}^{T}\left[ y_{t} \log \hat{y}_{t}+\left( 1-y_{t}\right) \log \left( 1-\hat{y}_{t}\right) \right] \end{aligned}$$

The model’s performance is evaluated through its capacity to generate syntactically valid molecules, that is, molecules that respect the SMILES notation rules for considering a molecule as synthesizable. This evaluation is performed using the RDKit tool after generating each instance. In addition to validity, it is important to assess whether the model can generate unique and novel instances compared to the training dataset. Furthermore, we employed the Tanimoto diversity metric to evaluate the molecular similarity in the set of generated molecules (internal) and in the training set (external). This metric involves obtaining the extended connected fingerprints (ECFP) of the molecules, which are bit vectors that contain structural and functional information about the compounds. To obtain these descriptors, the neighbourhood of each non-hydrogen atom of the molecule is analyzed up to a user-defined radius and mapped into integer codes using a hashing procedure [24].

### Pretraining of the biological affinity predictor

#### Datasets and preprocessing

The dataset used to train the Predictor includes the SMILES of the compounds and their corresponding biological affinity for the USP7 target. The parameter that measured the biological affinity was the negative logarithm of the half-maximal inhibitory concentration ($pIC_{50}$). Nevertheless, one of the problems related to the USP7 target is the scarcity of biological affinity information to train DL models properly. As a result, as the existing datasets contain few examples, it is challenging to implement robust models with generalization capability. In this case, the following approach was to add novel instances to the USP7 dataset. However, these novel instances relate compounds with the biological affinity for other targets that are similar to the USP7. In this sense, several targets were selected based on their catalytic domain similarity with the USP7. We assembled a final dataset containing 1453 molecules (see Table S2 of the Supporting Information for more details). The reasoning behind augmenting the dataset is that if the catalytic domains of two targets are similar, then the active groups of two molecules interacting with each target will also be similar. Still, despite the model being trained with the assembled dataset, we performed a fine-tuning step with the compounds especially related to the USP7 target after the initial training step. The objective was to achieve a valid training process and to create a model that could provide meaningful predictions for the USP7 target.

#### Model

We aim to obtain a model that learns to establish the relationship between chemical structures and their corresponding $pIC_{50}$ values. Therefore, it was analyzed the best approach to implement this model regarding the molecular descriptor and DL architecture. The studied combinations are summarized in Table 1. Following that, we have designed reward functions for each objective in order to map each predicted property to the respective reward based on how the property should be optimized (see Fig. S1 of the Supporting Information). Hence, it will be possible to explore the chemical space and identify the molecules with the highest potential to interact with the target.

**Table 1:** Tested models for the Predictor implementation

Model	Descriptor	DL architecture	Attention
A	ECFP4	Multi-layer FCNN	No
B	ECFP6	Multi-layer FCNN	No
C	RDKit fingerprint	Multi-layer FCNN	No
D	SMILES	RNN	No
E	SMILES	RNN	Yes
F	SMILES	Bidirectional RNN	No
G	SMILES	Bidirectional RNN	Yes
H	SMILES	Bidirectional RNN + RNN	No
I	SMILES	Bidirectional RNN + RNN	Yes
J	SMILES	Separated Bidirectional RNN	Yes

As previously stated, ECFPs are molecular descriptors that represent the structural features of molecules. Hence, these descriptors allow us to verify the presence or absence of substructures in molecules, making ECFPs well-suited for problems of biological activity prediction [25]. The radius used to analyze the neighbourhood of each atom can be adjusted, and, in this work, we tested ECFP4 and ECFP6 that apply radii of 2 and 3 atoms, respectively. Additionally, the RDKFingerprint implemented by the RDKit tool (http://www.rdkit.org/http://www.rdkit.org/) was also tested as the molecular descriptor. This algorithm starts by identifying all subgraphs in a molecule containing a number of bonds within a predefined range, then it applies a hashing procedure to the subgraphs and finally transforms it to generate a bit vector of fixed length.

One of the aspects that we aim to study in this model is the effect of attention mechanisms on the Predictor performance. Attention mechanisms were initially used in encoder-decoder models with recurrent architectures. The rationale associated with the application of attention is to be able to analyze a sequence and ensure that the output of this analysis has a direct influence on all elements of that sequence [26]. Without attention, the output is just the result of the last hidden state of the RNN, that is, all intermediate hidden states would be ignored. Although LSTM layers are able to capture long-range dependencies, for longer inputs, important parts may be overlooked using this architecture. Attention mechanisms were designed to address this problem since they manage to keep all the relevant information of the input in the context vector by assigning different relative importance to each processed element [26].

Hence, the attention capabilities can be applied to the Predictor implementation. In this scenario, rather than analyzing the significance of each word in the sentence, we aim to assess the relative importance of each molecular token to the $pIC_{50}$ value associated with the molecule. In practice, the RNN context vector for the molecule m ($C_m$) will be calculated as a weighted average of all preceding hidden states ($h_{i}$), as indicated in Eq. 2.

2 $$\begin{aligned} \begin{aligned}&C_{m}=\sum _{i=1}^{T} \alpha _{i} h_{i} \\&\alpha _{i}={\text {softmax}} \Big [ {\text {tanh}}\Big ( e (O_{m},h_{i}) \Big ) \Big ] \end{aligned} \end{aligned}$$

The weights $\alpha _{i}$ are the score calculated by a feedforward neural network (e) that extracts the alignment between the input token and the output ($O_m$). A hyperbolic tangent transformation, followed by a softmax activation, is applied to this score so that the values can be normalized between 0 and 1. Thus, the score has a dimension (T,1), and each element represents the importance of the token for the output.

### Conditioned molecular generation dynamics through RL

After pre-training the Generator and the Predictor, both models will be integrated into an iterative process comprising the generation and evaluation of compounds to identify an interesting set of hit compounds. Throughout this process, the Generator will be trained with RL using the reward assigned by the Predictor. The Predictor should indicate to the Generator the regions of the chemical space that have a higher probability of interacting with the USP7 target and adequate physicochemical properties.

Formally, the implementation of this method is based on the Markov Decision Problem (MDP) setting. In this work, the MDP setup was adapted to the molecular generative dynamics. The actions selected by the agent will be the sampling of new molecules by the Generator. Then, the external environment evaluating the actions will coincide with the Predictor’s evaluation of the molecular properties. The Generator will learn by experience throughout the process and the RL setting implemented in this work was the REINFORCE algorithm [27]. As a result, the policy used to select the actions corresponded directly to the weights of the Generator, which are updated through the gradient descent algorithm. The ultimate goal of this policy-gradient algorithm is to select the actions that maximize the scalar reward assigned to the agent, as stated in Eq. 3.

3 $$\begin{aligned} R_{t}=\sum _{k=0}^{T} \gamma ^{k} r_{t+k+1} \end{aligned}$$

where $R_{t}$ is the reward, *t* is the time step, *T* is the final time step, and $\gamma$ is a parameter ranging from 0 to 1, denoting how much the future reward is worth in the present. This methodology allows the Generator to learn the more appropriate behaviour through trial and error, that is, without applying supervised learning. In practice, the policy ($\pi$) will choose the next action based on the distribution of probabilities derived from the previous context. The objective of the RL training is to improve the policy successively, i.e., the parameters of the Generator.

The Generator starts the dynamics by sampling a batch of molecules. Afterwards, we analyze each molecule by disassembling it into its separate tokens. Each token represents a taken action, and the loss function evaluates the probability of choosing each action. At each step, the cumulative loss is calculated by summing the probability analysis for the batch of molecules. As described in Eq. 4, $R_t$ is the reward assigned to the Generator by selecting the action $A_t$ when it was in the state $S_t$, following the policy parameters $\theta _t$, where $\alpha$ is the learning rate. This reward value is multiplied by the natural logarithm of the corresponding probability of taking that action, allowing the agent to learn which actions should be chosen more or less frequently in future similar situations. Thus, whenever the agent returns to the same state throughout the generation process, it will select the actions that previously brought it more reward and avoid the others. After following this procedure for the batch of molecules, the gradient of the loss function is computed, and the weights of the Generator are updated accordingly to minimize the loss function.

4 $$\begin{aligned} \theta _{t+1}=\theta _{t}+\alpha \gamma ^{t} R_{t} \nabla \ln \pi \left( A_{t} \mid S_{t}, \theta _{t}\right) \end{aligned}$$

### Self-adaptive molecular optimization strategy

USP7 is a cysteine protease that is involved in several signalling pathways. Its substrates and binding partners influence functions such as cell cycle regulation (e.g. cyclins), transcription factors (e.g. c-jun, NF-KB), immunological response, tumour suppression, epigenetic control, and DNA repair [28]. Therefore, this protein plays an important role in crucial post-translational mechanisms for cell dynamics, specifically through ubiquitination/deubiquitination in the p53-MDM2 pathway, which has been linked to cancer-protection mechanisms [29, 30]. Furthermore, the USP7 gene was found to be overexpressed in various cancers, which generally indicates poor tumour prognosis [28]. For this reason, small molecules that can inhibit the USP7 target may function as anticancer agents with significant therapeutic potential. Despite this possible impact, it has not yet been possible to identify a sufficiently effective and selective USP7 inhibitor capable of reaching the late phase of clinical trials.

In this work, it is expected that the generated molecules have the potential to inhibit USP7. Therefore, the objective is to maximize the $pIC_{50}$ of the generated molecules and to promote the inhibitory interaction between the sampled molecule and the catalytic domain of the USP7 target. Nonetheless, during the generation of the molecules, another property will be considered to ensure that the identified hits are drug-like and synthesizable in the laboratory. In other words, we intend to optimize a property that considers factors such as large rings, non-standard ring fusions, stereocomplexity, and molecule size throughout the synthesis process. This property is the synthetic accessibility score (SAS), and it condenses the evaluation of the mentioned properties into a single scalar ranging from 1 (easy to synthesize) to 10 (impossible to synthesize) [31].

Thus, the computational task is to maximize the $pIC_{50}$ while minimizing the SAS of the generated molecules. In the RL setting, this scenario can be seen as the optimization of two potentially competing reward values, i.e., the Generator should learn how to select actions that assure the optimization of both properties at the same time. The followed approach was to design a single reward function composed of these two terms. Therefore, the multi-objective formulation of the problem is transformed into a single-objective optimization task by constructing a single, scalar, additive reward function that considers the influence of both parameters. In this scenario, each objective is associated with a weight (*w*) that indicates the relative importance attributed by the RL agent. This numerical weight is assigned beforehand, and it can be arbitrarily selected as long as each weight ranges between 0 and 1 and the sum of all assigned weights equal 1. There are several strategies to implement this method that vary according to the specific problem and user’s preferences. This work provides an intuitive method for determining these preferences based on the individual rewards obtained during the optimization process. The first step is to normalize the rewards associated with each property between 0 and 1 to compare each objective’s optimization level. We then determine the initial weight assignment according to the desired preference. From this starting point, the weights are updated, taking into account the rewards registered for each property: if one objective becomes more favoured by the Generator than the other, the algorithm will adjust its weights to reverse this tendency and preserve the fair balance between the two. This self-adaptive method is based on the analysis of the last reward (*f*) and also on the variation ratio of the reward in a five-batch window (*VR*(*f*)). Pseudocode 5 describes the rationale for updating the preference weights throughout the optimization method.

5 $$\begin{aligned} \begin{aligned}&\textrm{f}(x)= w_{1}\mathrm {f_{1}}(x)+w_{2} \mathrm {f_{2}}(x)\\&\,\,\texttt {if}\,\, (w_{1} \cdot \mathrm {f_{1}}(x)>w_{2} \cdot \mathrm {f_{2}}(x)) \,\, \texttt {and} \,\, VR(f_{2})<0.05 \\&\qquad w_{1}=w_{1}-0.1; \quad w_{2}=w_{2}+0.1; \\&\,\,\texttt {elif} \,\,(w_{1} \cdot \mathrm {f_{1}}(x)<w_{2} \cdot \mathrm {f_{2}}(x)) \,\, \texttt {and} \,\, VR(f_{1})<0.05\\&\qquad w_{1}=w_{1}+0.1; \quad w_{2}=w_{2}-0.1; \end{aligned} \end{aligned}$$

It should be stressed that in real-world problems with competing objectives to consider, it is impossible to find an ideal solution that optimizes both. Hence, it is necessary to determine a compromise that meets the demands of the task. In this context, the solutions obtained in the optimization process correspond to molecules. We employed the Pareto diagram visualization tool to visualize and help identify the most promising molecules. It is a method for representing a set of solutions that describes the trade-off between competing objectives [32]. This diagram distinguishes between non-dominated and dominated solutions. The former solutions have all the objectives better optimized than any dominated solution, i.e., all dominated solutions are worse than the non-dominated ones considering each objective. After identifying the non-dominated solutions, a comparative analysis will be performed to determine the most promising molecular hits based on the biological affinity for the target and its synthesizability.

### Molecular docking calculations

The crystal structure of the human USP7 was retrieved from the Research Collaboratory for Structural Bioinformatics (RCSB) Protein Data Bank (PDB) database (PDB ID: 5NGF) with a resolution of 2.33 Å elucidated through X-ray crystallography. Employing Molecular Operating Environment (MOE) software package (v. 2022.02) [33], chain B, the respective crystallographic ligand, 1,2-ethanediol and water molecules were removed from the system. The receptor structure (chain A) was protonated (at pH 7.4 and 310.15 K) using the Protonate 3D tool, and hydrogen atoms were added. Amber10:EHT forcefield was used to assign atom types in the receptor structure, which was further energy minimized using the same forcefield. Using the triangle matcher method in the placement phase and rigid receptor for refinement, self-docking experiments were conducted to predict the best scoring functions from all the available scoring functions in MOE. The best self-docking results were achieved for the GBVI/WSA dG scoring function in both the placement and refinement stages. These settings reproduce the binding pose of the co-crystallized ligand (8wn) in the USP7 crystal structure with a root-mean-square deviation (RMSD) of 1187 Å. Non-docking calculations of the dataset were performed. The ligands were prepared using Amber10:EHT forcefield to assign atom types and protonated at pH 7.4 and 310.15 K with the Protonate 3D tool, followed by energy minimization. The prepared dataset was docked into the catalytic domain of USP7, directing the docking for the crystallographic ligand atoms site. Thirty poses were generated for each molecule using the triangle matcher method in the placement stage and scored by the GBVI/WSA dG scoring function. Subsequently, the five best-scored poses were submitted to rigid receptor refinement using GBVI/WSA dG scoring function. After the docking simulations, the compounds were sorted according to their docking scores. The binding poses and the protein-ligand interactions of the top-ranked compounds were visually inspected. Images of the compound’s interactions with USP7 and surrounding residues were produced using MOE (v. 2022.02) software.

## Results and discussion

### Performance of the pre-trained generator

The evaluation of the pre-trained Generator aims to determine whether the model was able to learn the chemical rules for constructing molecules based on the SMILES notation through the generation of new molecular instances. Additionally, it is also crucial to verify other properties, such as molecular diversity and the novelty rate of the new molecules sampled. We have employed the Guacamole benchmark framework to rigorously evaluate these essential aspects of the unbiased Generator. This quantitative assessment allows us to gauge the performance of our model and compare it to the current state of the art. In this sense, 10,000 molecules were generated, and properties such as the rates of valid, novel and unique molecules were evaluated, as well as the Fréchet ChemNet Distance (FCD) and Kullback–Leibler divergence (KLD) [34]. We also compared the pre-trained Generator with the most well-known molecular generative models, including SMILES LSTM [35], VAE [18], AAE [36], Graphs with Monte Carlo Tree Search (MCTS) [37] and ORGAN [16]. The results are summarized in Table 2, with the best approaches for each metric highlighted in bold.

**Table 2:** Evaluation of pre-trained and optimized Generators and comparison with the state-of-the-art approaches

Method	pIC50	SAS	Validity	Uniqueness	Novelty	FCD	KLD
Unbiased	5.27 ± 0.62	2.55 ± 0.64	0.966	0.984	0.982	0.905	0.914
Biased	**6.35 ± 0.41**	**2.03 ± 0.26**	0.918	0.956	0.913	**0.926**	0.956
ORGAN	–	2.51 ± 0.65	0.379	0.841	0.687	0.000	0.267
VAE	–	2.76 ± 0.71	0.870	0.999	0.974	0.863	0.982
AAE	–	2.43 ± 0.47	0.822	**1.000**	**0.998**	0.529	0.886
Graph MCTS	–	2.72 ± 0.82	**1.000**	1.000	0.994	0.015	0.522
SMILES LSTM	–	2.65 ± 0.67	0.959	1.000	0.912	0.913	**0.991**

The high validity rate suggests that the unbiased Generator has effectively learned the fundamental principles for building molecules using SMILES notation. Simultaneously, the small values of FCD and KL divergences demonstrate that the unbiased Generator model has successfully captured the underlying molecular distribution within the training data. Furthermore, the model’s ability to generate novel and unique instances is demonstrated by the high levels of novelty and uniqueness, respectively. Therefore, the results allow us to conclude that the pre-trained Generator reflects a promising compromise between learning to construct molecules based on the training dataset and exploring new regions within the chemical space. Also, it is worth highlighting the similarity between the achieved results by the proposed models and state-of-the-art approaches, particularly in terms of the novelty of the generated molecules. These findings are particularly encouraging since the non-optimized Generator is the starting point for all subsequent experiments.

### Performance of the predictor

The Predictor was implemented to map the SMILES of a compound to its capacity to inhibit the USP7 target. This inhibitory potency was estimated using the $pIC_{50}$ metric. Thus, the objective was to implement a regressive model that minimizes the distance between the actual $pIC_{50}$ of the molecules and the value predicted by the model by minimizing the mean-squared error (MSE). In the generation dynamics proposed by this work, the role of the Predictor is fundamental because it is this model that will guide the process of exploring the chemical space of the Generator. In other words, the zones indicated by the Predictor as having the highest reward will be more thoroughly explored by the Generator, and, as a result, it is important that the model learns to identify these zones with the preceding training process.

It should be emphasized that for each model mentioned in Table 1, we studied the best parameters to establish the configuration that would extract the best performance from each architecture. Although different versions of Predictor have been implemented, some aspects of its training process have been established for all. The data division was performed using cross-validation to identify the best configuration, and the molecules were split into five folds. For each fold, 85% of the compounds were employed in training and the remaining 15% as validation. A hold-out set was also defined to verify the performance of the models in previously unseen instances. Regarding the final model, the applied optimizer was Adam, and the model was trained during 50 epochs. Nonetheless, in order to mitigate potential overfitting, two callbacks were applied during the training process, namely, early stopping with a patience of 15 epochs without improvements and also model checkpoint. Additionally, we applied a method to standardize the $pIC_{50}$ values using the interquartile range to ensure a faster and more accurate convergence of the learning process.

**Fig. 2:**
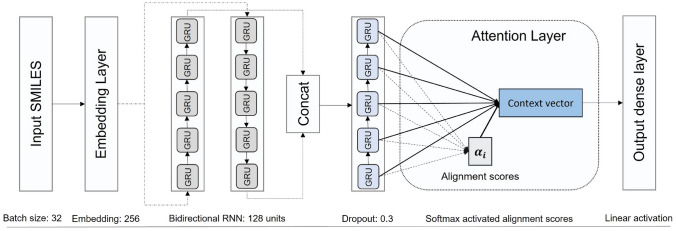
Configuration I Predictor. Identification of the RNN-based architecture with an attention layer before the output

As indicated in 1, configuration *I* employed SMILES notation and an attention mechanism in its architecture to predict the $pIC_{50}$. The model consisted of an embedding layer, followed by a bidirectional gated recurrent unit (GRU) layer and another GRU layer. Then, attention was applied to the hidden states obtained from the GRU layer before the dense output layer. As indicated in Fig. 2, the dimension of the embedding vectors was 256, and 128 units were used in the layers with recurrent architectures. A dropout of 0.3 was applied to minimize overfitting, and a dense layer linearly activated was applied as the output layer. The role of the bi-directional GRU layer is critical for an efficient feature extraction procedure. It combines forward, and backwards hidden layers to be able to access the sequence’s preceding and succeeding contexts. Nevertheless, while this architecture provided the best result, it is clear that the performance might be improved if more examples were used to train the model.

**Fig. 3:**
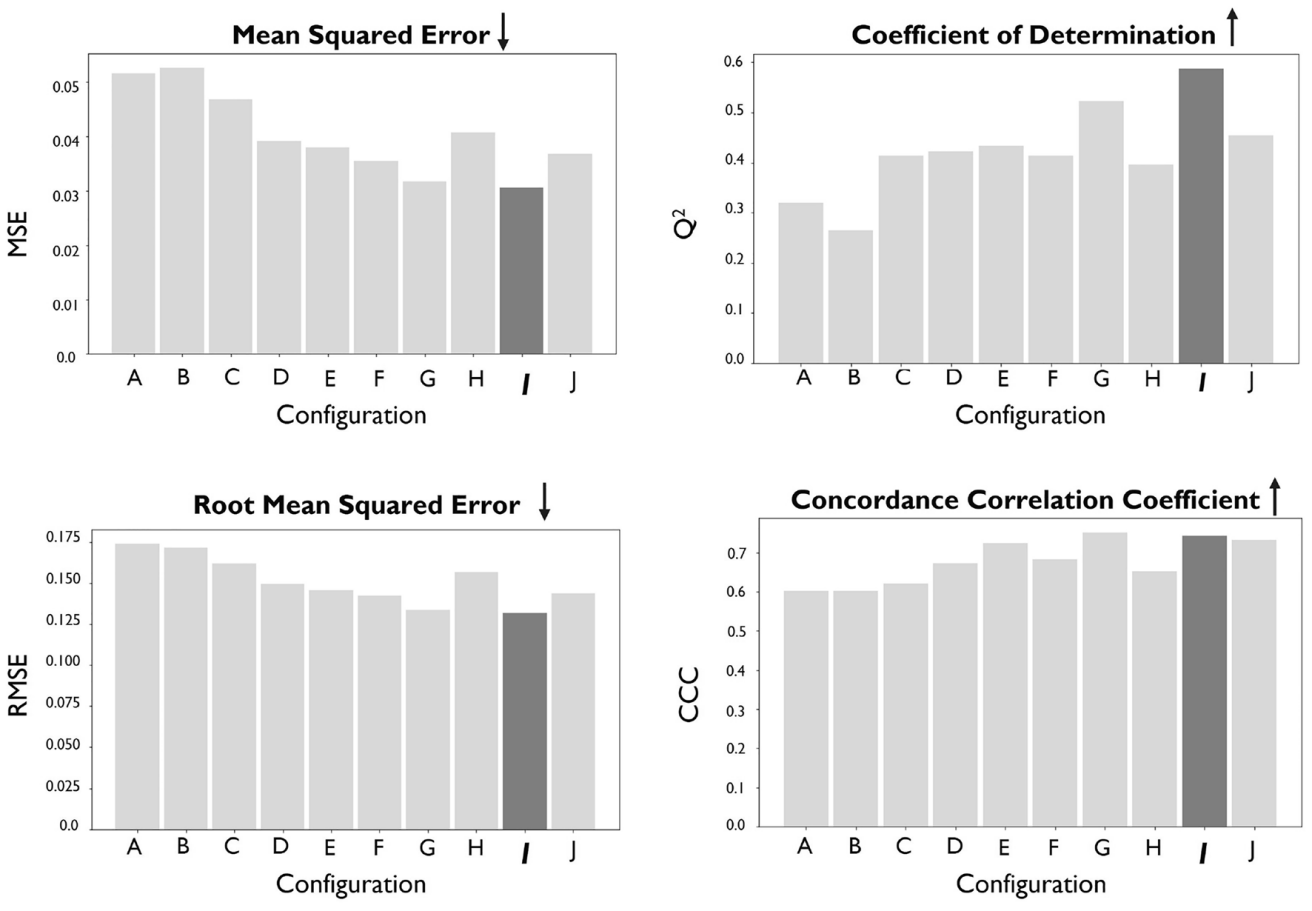
Results Predictor. Identification of the best model configuration through the evaluation of MSE, $Q^2$, RMSE, and CCC

The metrics used to analyze the performance of the Predictor are intended to evaluate the quality of the implemented regression. Thus, in addition to the MSE, we evaluated the coefficient of determination ($Q^2$), root-mean-squared error (RMSE), and concordance correlation coefficient (CCC). Figure 3 shows the obtained results for each implemented Predictor.

**Fig. 4:**
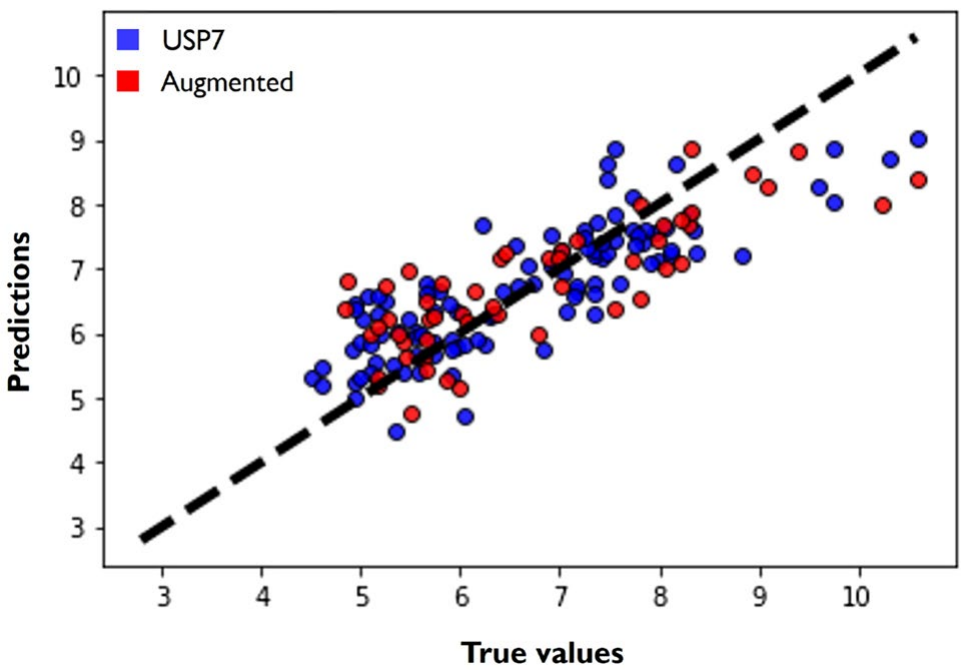
Predictor evaluation. Predictions for the hold-out set against the true $pIC_{50}$ values. The diagonal line (predicted = true value) illustrates what would be the perfect model. The color of each point indicates whether the instance is a true USP7 instance (blue) or whether it was obtained through the data augmentation strategy (red)

Figure 3 depicts a significant difference in the performance between models implemented using SMILES and those trained with fingerprint-based descriptors. Predictors built with ECFP4, ECFP6, and RDKit fingerprints perform worse when analyzing all metrics, that is, they are less informative descriptors when compared with raw SMILES. Another interesting aspect of this study is the fact that models containing the attention mechanism in their architecture outperform similar models without attention. This finding confirms the benefits of this conceptually simple yet effective DL layer. In this scenario, it is noticeable that the AM helps the Predictor identify the tokens in the molecule that will be decisive for determining whether or not an interaction can occur.

Additionally, it is possible to confirm that configuration *I* provided the best results. Figure 4 depicts the predictions of the model against the true $pIC_{50}$ values for the hold-out set, where it is possible to observe a considerable density around the reference line for all ranges of $pIC_{50}$.

### Conditioned molecular generation with optimized properties

The conditioned generation of compounds was based on the application of an RL optimization procedure combined with a self-adaptive method of updating the preferences associated with each objective. Before obtaining the Pareto front disposing of the solutions, it was necessary to determine the best hyperparameters for applying the framework. Table 3 indicates the optimal values obtained for the number of epochs, softmax temperature, initial weight assignment, and the weight update step to apply throughout the process. We implemented a grid search to find the most suitable model configuration for biasing the two properties in the desired direction while maintaining the validity rate of the SMILES. The defined search spaces as well as the results of all experiments, are shown in Table S3 of the Supporting Information.

**Table 3:** Implementation parameters of the self-adaptive conditioned generation framework

Epochs	Softmax Temp.	Inital weights ($pIC_{50}$, SAS)	Weights update step
90	0.80	[0.6, 04]	0.05

**Fig. 5:**
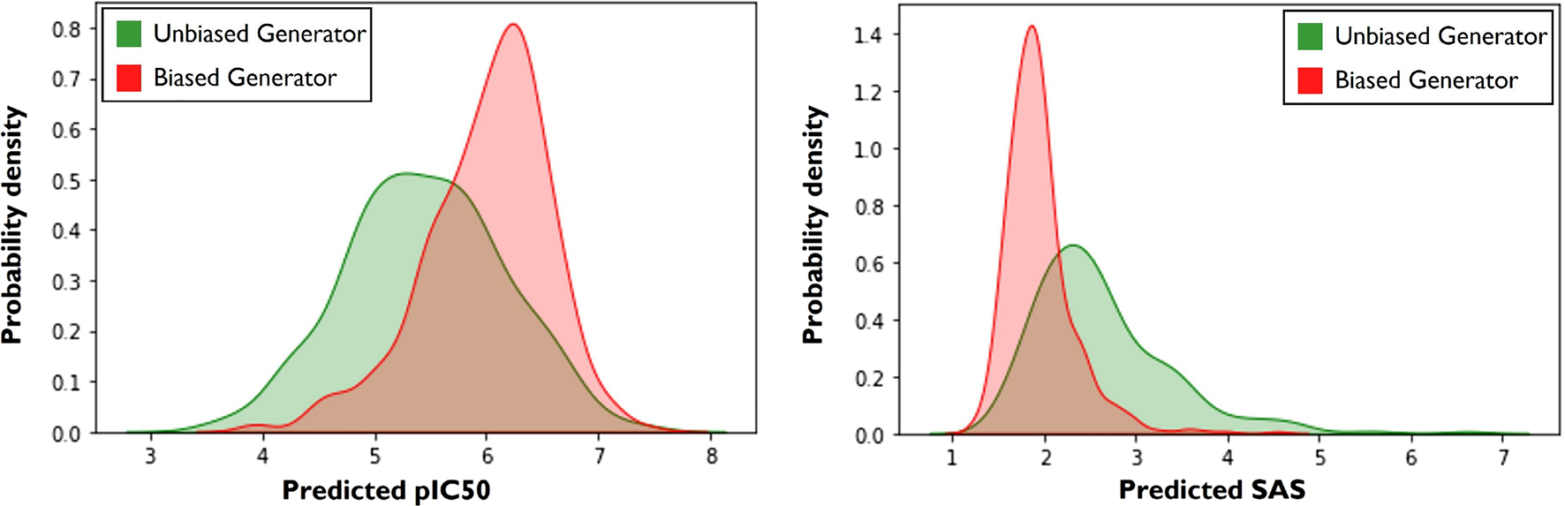
Biased Generator evaluation. Comparison of $pIC_{50}$ and SAS from molecular samples obtained by the pre-trained Generator (unbiased) and optimized Generator (biased)

We applied the optimal configuration to re-train the Generator with the policy-gradient algorithm. The obtained policy generated molecules with high biological affinity for the USP7 target and the SAS biased as desired. To demonstrate the effect of the RL training procedure, we have generated 200 molecules with both the optimized and the non-optimized Generators. Figure 5 illustrates the transformations in the distributions for both molecular properties.

**Fig. 6:**
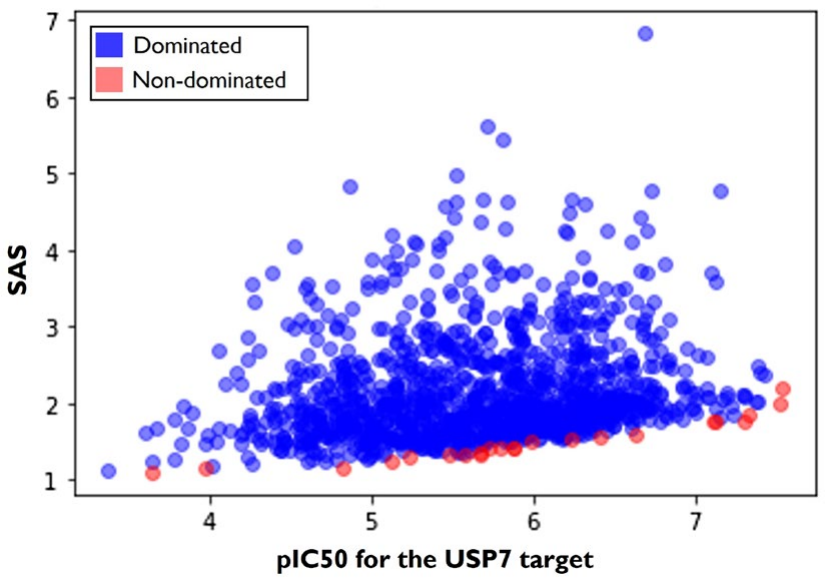
Pareto front. Representation and identification of dominated and non-dominated solutions

Afterwards, the objective was to identify specifically the best molecular hits generated by the optimized policy, considering the trade-off between the two properties. As represented in Fig. 6, the Pareto Front was designed to identify the most promising non-dominated solutions. The non-dominated solutions, described in red, were identified, and their molecular properties were evaluated more thoroughly as they were the solutions that provided the best compromises between the optimization of $pIC_{50}$ and SAS.

### Analysis of the optimized compounds

**Fig. 7:**
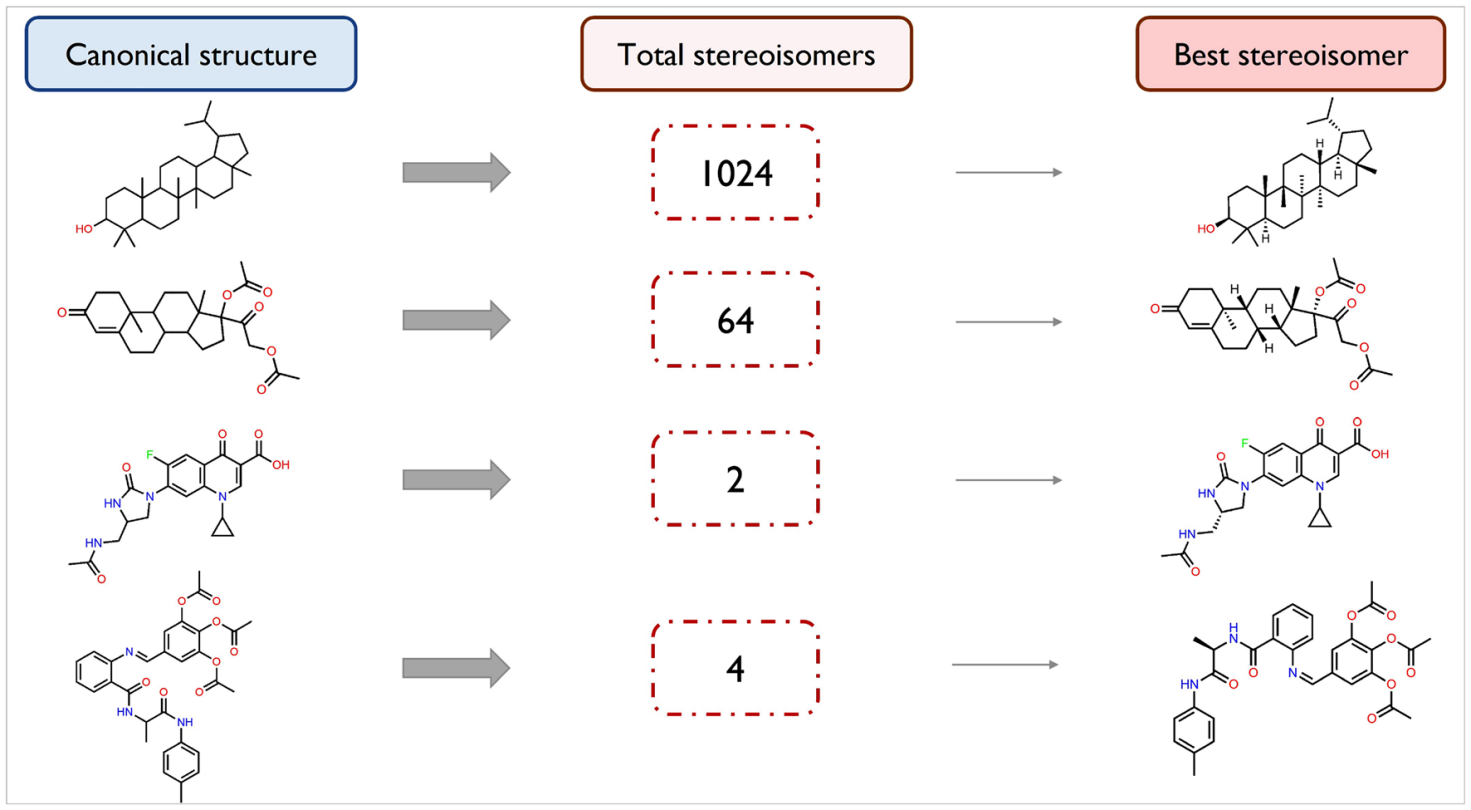
Stereochemistry of the generated molecules. Enumeration of the all stereoisomers for each hit candidate and identification of the best spatial conformation of atoms

One of the purposes of this work was to discover compounds with the potential to become promising hits while including information about the spatial arrangement of their atoms. In fact, this information is essential for the biological activity of the molecule and for the presence or absence of potentially undesired effects. Although some drug-like molecules don’t have any stereoisomers or the most suitable conformation is evident, there are examples where the indication of stereochemistry is absolutely fundamental for understanding the potential of the candidate drug. When the molecules have a natural origin and contain multiple chiral centres, the stereochemical information plays a relevant role. In this sense, the goal was to use the canonical SMILES of each previously identified molecular hit to determine the corresponding stereoisomer with the most promising inhibitory potential. The Predictor was developed for this purpose since the training compounds contained stereochemical information and the applied SMILES vocabulary included the symbols required to represent this chemical aspect. Therefore, an RDKit method was used to enumerate all the possible stereoisomers from each previously identified candidate hit with the Pareto diagram, allowing the Predictor to identify the most suitable configuration. Figure 7 illustrates this process for some examples. The number of stereoisomers obtained for each candidate hit depends on the number of chiral centres of its molecular structure (c) under the condition of $2^c$. For this reason, hit compounds with multiple chiral centres can have a wide variety of stereoisomers. Although the proposed method intends to identify the best configuration in terms of biological affinity, the indicated compound may be unrealistically difficult to synthesize. Hence, when selecting the best molecular hits, it is critical to take into account the trade-off between biological affinity and synthesizability of each stereoisomer.

**Fig. 8:**
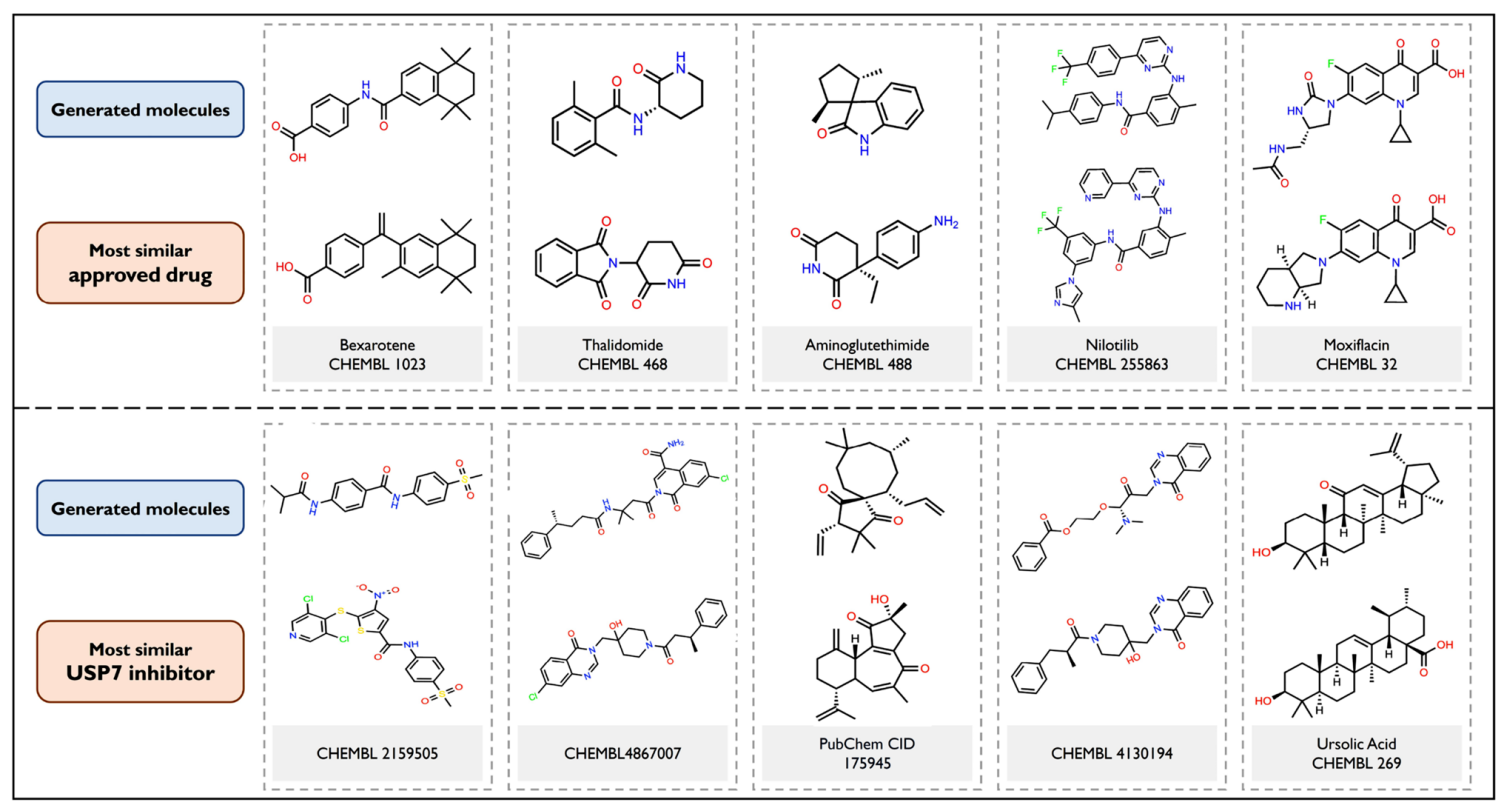
Molecular similarity analysis. Comparison of generated molecules with known anti-cancer compounds using Tanimoto similarity ($T_S$) and Tanimoto maximum common substructure ($T_{mcs}$)

Demonstrating the drug-like properties of the generated compounds is a fundamental step for validating the implemented method. One of the ways to demonstrate the quality of the generated hits is by showing their similarity to existing drugs, i.e., molecules which have already passed the tight sieve of evaluations to become approved drugs. In this work, we applied two metrics to assess molecular similarity: Tanimoto distance and Tanimoto maximum common substructure. Both are chemically intuitive methods for measuring the similarity between molecules, but the latter is useful to compare bioactive molecules since it allows the identification of local similarities within the molecular structures. Figure 8 demonstrates the similarities between generated molecules and known anti-cancer drugs. For each pair of molecules, it is noticeable a high similarity between their scaffolds, even analyzing the potential active sites of the compounds. These findings demonstrate that the Generator could explore pharmacologically interesting regions of the chemical space.

**Fig. 9:**
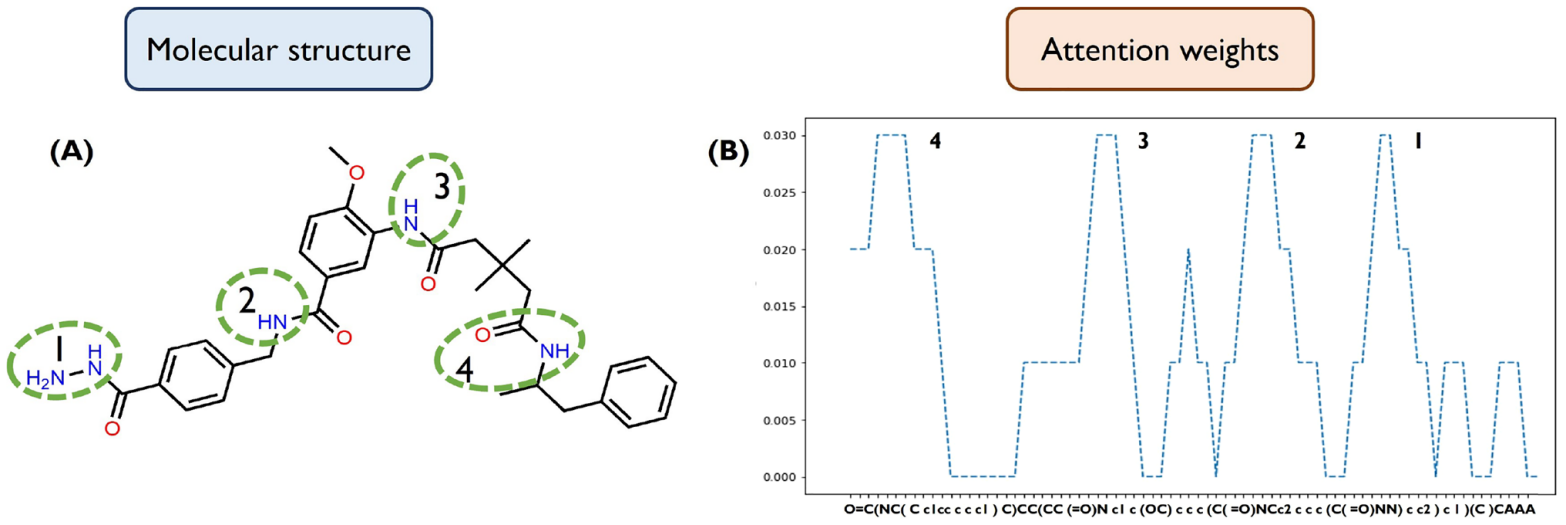
Attention analysis. **A** Visualization of regions considered as most important for the Predictor. **B** Attention weights across the molecular structure

**Fig. 10:**
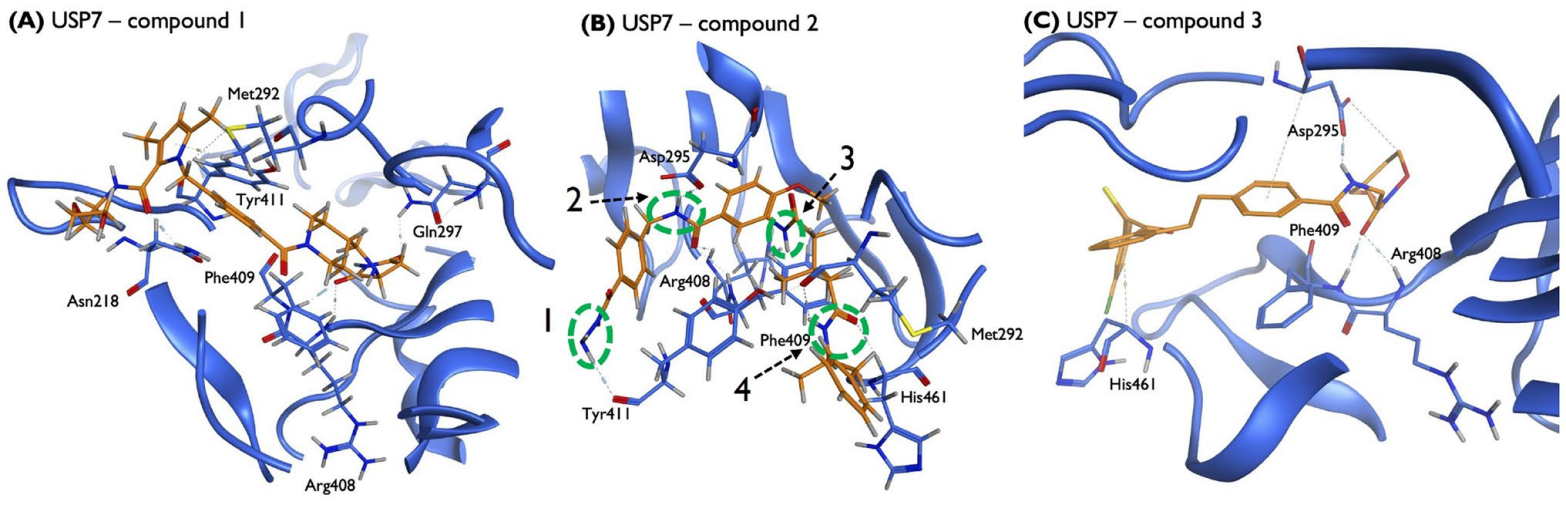
Schematic representation of three of the most representative protein-ligand interactions predicted by the molecular docking simulations for the novel generated molecules with USP7 catalytic domain. **A**, **B** and **C** Two-dimensional compound interactions and surrounding residues represented according to their chemical properties

The model’s explainability is another crucial factor for the validation of the molecular generative framework. In this context, explainability implies specifying the parts of the molecules that the model considers as most important to determine the affinity with the target, i.e., which active groups will be directly involved in the drug-target interaction. Herein, this analysis is possible by exploring the potential of the AM embedded in the Predictor’s architecture, specifically through the analysis of the attention scores. According to the output, the AM was trained to adjust the context vector computed by the recurrent architecture. The attention weights perform this adjustment since they are calculated to express the relative importance of each token to the $pIC_{50}$ of the molecule. We analyzed the attention weights across the structure of a sampled molecule, and the result is depicted in Fig. 9A. Additionally, it was performed a simulation of the interaction of this structure with the USP7 enzyme through molecular docking (Fig. 10B) to compare the findings. According to the distribution of the weights described in Fig. 9B, the molecule exhibits four essential subregions for the interaction. We highlight these molecular subregions through the dashed green. The functional group 1 is hydrazine, while 2, 3 and 4 are amides. As a result, Predictor considers these four regions as important functional groups in interacting with USP7. These assumptions were later confirmed by the docking simulation. Figure 10B depicts the ligand in yellow, with the nitrogen atoms in blue and the oxygen atoms in red. The green dashed line indicates the linguistic functional groups involved in the interaction. The interaction analysis reveals that these functional groups are the same as those previously identified in Fig. 9, which demonstrates that the attention spikes clearly identify FGs that establish interactions with key amino acids of the USP7. Thus, the Predictor’s ability to detect these potential interaction zones was confirmed, as they were also identified in the drug-target binding process indicated by the docking simulation.

### Virtual screening validation

**Fig. 11:**
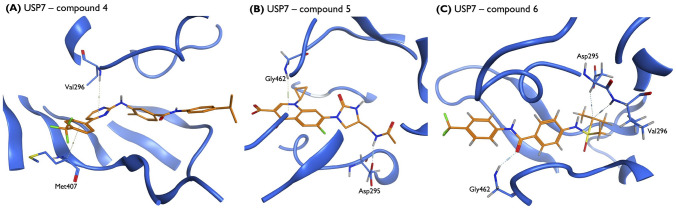
Schematic representation of three of the most representative protein-ligand interactions predicted by the molecular docking simulations for the generated molecules with scaffolds similar to known anticancer drugs with USP7 catalytic domain. **A**, **B** and **C** Two-dimensional compound interactions and surrounding residues represented according to their chemical properties

The virtual screening protocol was implemented to demonstrate the potential of the generated molecules to inhibit USP7 enzymatic activity. Molecules capable of interacting with one or more key residues of the catalytic domain, such as aspartic acid 295 (Asp295), valine 296 (Val296), glutamine 297 (Gln297), arginine 408 (Arg408), phenylalanine 409 (Phe409) and tyrosine 465 (Tyr465), were privileged due to their key role stabilizing the inhibitor within the binding pocket. The molecular docking simulations retrieved some compounds with a docking score comparable to that of the crystallographic inhibitor (S = − 10,1878). Compounds with lower docking scores were predicted to establish stronger interactions with USP7 on specific binding sites and, therefore, considered potentially good inhibitors of USP7 enzymatic activity. Figures 10 and 11 uncover three of the most representative protein-ligand interactions predicted by molecular docking calculations for the novel generated molecules and for the generated molecules with scaffolds similar to known anticancer drugs, respectively. As illustrated, hydrogen bonds and polar hydrogen-pi interactions were established between the ligands and key amino acid residues, including Gln297, Asp295, Arg408 and Phe409. Hydrogen bonds are established when a hydrogen atom bonded to a strongly electronegative atom interacts with another electronegative atom with a lone pair of electrons, creating a fundamental attractive force that will actively contribute to the interaction. In the polar hydrogen-pi interactions, the polar hydrogen atoms bonded to strongly electronegative atoms interact with aromatic molecules and conjugate pi-groups, creating a comparable or even larger attractive force than common hydrogen bonds. These results demonstrate the model’s potential to generate compounds that can establish strong interactions with key amino acid residues for the USP7 catalytic activity, potentially blocking the combination of ubiquitin with USP7 and revoking USP7 enzymatic activity.

## Final remarks

The obtained results suggest that, besides possessing a high biological affinity for the target, the molecules are synthesizable, drug-like, and contain relevant stereochemical information. Furthermore, the framework was able to indicate which parts of the compounds are most likely to be directly involved in the drug-target interaction. The validation of the proposed model was supported by the significant similarity of the small molecules generated compared to the existing USP7 inhibitors and with the insightful docking results.

One of the advantages of this framework is its versatility, which allows it to be easily adaptable to other targets or drug development tasks. The exclusive application of raw descriptors, such as SMILES notation, is an eloquent demonstration of this versatility. The applied deep learning architectures were able to extract the most pertinent information from this sequential input, allowing for the generation of molecules with bespoken properties. As a result, the reliability of the conditioned generation dynamics proves that both the Predictor and the Generator have learned non-abstract features about the chemical space and the USP7 target.

The stereochemistry and synthetizability of the compounds were also key elements addressed in this work. Although it is essential for some molecules to specify the relative positions in the space of their atoms, this information is often disregarded in computational generation frameworks. In this work, we sought to integrate stereochemical information in the most promising candidate hits to avoid situations where canonical SMILES can have two or more stereoisomers with differing biological characteristics or even harmful side effects. On the other hand, we worked to generate molecules that can be synthesized in the laboratory, and this objective was incorporated into the deep reinforcement learning setting alongside the drug-target biological affinity. The proposed strategy was to implement a self-adaptive linear weighted sum method to dynamically condense the influence of the two competing objectives into a single reward function. The self-adaptive nature of the weights selection allowed the RL agent to consider both objectives fairly throughout the optimization process and to determine the most suitable solutions according to the user’s preferences.

The Predictor architecture contained an embedded AM that identified sub-molecular regions with varying degrees of importance for the interaction between the candidate drug and the target. The AM proposed by Bahdanau et al. has been applied with recurrent architectures to help them learn the temporal dependencies present in molecular structures. While RNNs such as LSTM can understand these dependencies, the longer the sequences of atoms, the more difficult it is to learn them correctly. In this case, the AM technique was applied to learn a set of attention weights that conditioned the context vector of the RNN architecture according to the target, which in this task was the biological affinity. Attention coefficients represent varying degrees of importance for different sections of the molecule, allowing the model to locate the key active sites. This information is helpful for the implemented framework as it enables a more focused and orientated exploration of the regions of the chemical space that are important for the target. Also, the visualization of the attention coefficients enabled the validation of the Predictor’s robustness since the model was able to identify with great precision the critical zones of the molecule. Hence, in addition to confirming the potential inhibitory interaction between the best compounds and the USP7, the docking experiments corroborate the accurate identification of the molecular regions most involved in this interaction by the Predictor. Identifying these zones is also essential in the subsequent steps of optimization of the candidate drug because it indicates which regions of the molecule can be altered to ensure the desired physicochemical properties and which parts cannot be altered as they are strongly related to their biological affinity.

Despite the encouraging results obtained, the number of molecules used to train the Predictor was restricted due to the number of USP7 inhibitors reported in the literature thus far. The main advantage of the DL models is the capacity to extract hidden patterns from many examples. Therefore, it would be important to assemble a dataset with more molecular diversity to exploit the potential of the architecture in $pIC_{50}$ prediction fully. Although we gathered biological affinities of targets with high similarity in the catalytic domain compared to the USP7 target, this data augmentation step may introduce dubious information into the model. Nevertheless, the selected option optimized the threshold between the quantity of data and the selectivity of the compounds against the USP7 target.

## Conclusions

The implemented model allowed us to generate potentially inhibitory hit molecules for the USP7 target. We applied deep learning architectures to streamline the early steps of the drug development process while also addressing critical issues of current computational generative state-of-the-art models. Hence, it was possible to implement a robust, self-explanatory, and useful de novo deep generative framework to identify putative inhibitors for a target with high biological interest, such as USP7. We expect this work to be a step forward to making USP7 inhibition an alternative and effective therapeutic pathway in cancer. Also, we demonstrated that it is possible to apply the capabilities of attention to identify the most promising regions of chemical space, allowing to shorten the period of time and the efforts required to discover new bioactive compounds of interest.

### Supplementary Information

Below is the link to the electronic supplementary material.

Electronic supplementary material 1 (PDF 471 kb)

## Data Availability

The data and source code used in this study are available at: https://github.com/larngroup/targeted_generation_stereo
