# Supplementary material for: Artificial intelligence for prediction of biological activities and generation of molecular hits using stereochemical information
Source: J Comput Aided Mol Des. 2023 Oct 17;37(12):791–806. doi: 10.1007/s10822-023-00539-9 (PMC10618333; doi:10.1007/s10822-023-00539-9)
Supplement: Supplementary file 1 — Electronic supplementary material 1 (PDF 471 kb) [file 10822_2023_539_MOESM1_ESM.pdf]

Supporting Information

Artificial Intelligence for Prediction of Biological Activities and Generation of molecular hits using Stereochemical Information

Tiago O. Pereira[1], Maryam Abbasi[1], Rita I. Oliveira[2,3],

Romina A. Guedes[2,3], Jorge A. R. Salvador[2,3] and Joel P. Arrais[1]

[1] Centre for Informatics and Systems of the University of Coimbra, Department of Informatics Engineering, Univ Coimbra, Coimbra, Portugal,
[2] Laboratory of Pharmaceutical Chemistry Faculty of Pharmacy, Univ Coimbra, Coimbra, Country,
[3]Centre for Neuroscience and Cell Biology Centre for Innovative Biomedicine and Biotechnology, Univ Coimbra, Coimbra, Portugal

Supplementary Tables

Parameter	Value
**Units**	512
**Epochs**	16
**Batch size**	16
**Optimizer**	RMSprop
**SMILES encoding**	Embedding
**Learning rate**	0.001
**Dropout rate**	0.2
**Sampling temperature**	0.75

Table S1 - Implementation parameters for Generator pre-training step.

Target	Compounds
**USP-7**	449
**USP-18**	2
**Acetyl-CoA carboxylase 1**	394
**USP-1**	4
**USP-47**	2
**Cystathionine beta-synthase**	14
**USP-9X**	12
**BAP-1**	21
**UDP-N-acetylmuramoylalanine--D-glutamate ligase**	7
**USP-13**	1
**E3 ubiquitin-protein ligase UHRF1**	21
**USP-28**	162
**USP-5**	1
**USP-25**	48
**USP-30**	407
**UCH-L3**	6
**USP-14**	1
**Beta-glucosidase cytosolic**	3
**Prolyl-tRNA synthetase**	27
**D-alanyl-D-alanine carboxypeptidase**	24
**Salivary alpha-amylase**	28

Table S2. Composition of the dataset assembled to train the Predictor in terms of target and the respective number of compounds

Run	Iterations	Initial weights (USP7, SAS)	Temperature	Weight step	Valid	Unique	Sas			pIC50 USP7		
							Min	Avg	Max	Min	Avg	Max
**1**	75	[0.5,0.5]	0.75	0.05	95	100,00	1.265	2,197+-0.724	5.079	4.087	5.639+-0.679	7.741
**2**	75	[0.5,0.5]	0.75	0.1	95.5	100	1.326	2.579+-0.773	5.705	3.969	5.857+-0.635	7.685
**3**	75	[0.5,0.5]	0.8	0.05	85	97.65	1.097	1.873+-0.478	4.240	3.643	5.647+-0.705	7.082
**4**	75	[0.5,0.5]	0.8	0.1	78	98.718	1.455	2.485+-0.924	5.335	3.764	5.896+-0.746	7.812
**5**	75	[0.6,0.4]	0.75	0.05	75.0	99.33	1.205	1.916+-0.465	4.054	1.205	5.2+-0.615	7.017
**6**	75	[0.6,0.4]	0.75	0.1	94	99.47	1.097	2.264+-0.801	4.969	3.901	5.467+-0.695	7.148
**7**	75	[0.6,0.4]	0.8	0.05	97	78.35	1.497	2.695	4.726	4.219	5.888+-0.565	7.189
**8**	75	[0.6,0.4]	0.8	0.1	98	100	1.167	2.150+-0.557	4.554	3.661	5.462+-0.660	7.274
**9**	75	[0.7,0.4]	0.75	0.05	96	98.96	1.400	2.553+-0.642	4.949	4.177	6.121 +-0.531	7.386
**10**	75	[0.7,0.4]	0.75	0.1	99.5	25.63	1.412	1.813 +-0.277	2.511	5.058	6.063 +-0.508	7.379

**11**	75	[0.7,0.4]	0.8	0.05	84	100	1.289	2.378 +-0.676	2.378	4.108	5.719 +-0.659	7.149
**12**	75	[0.7,0.4]	0.8	0.1	95.5	43.455	1.459	2.627 +-0.452	3.461	4.445	6.193+-0.743	7.671
**13**	100	[0.5,0.5]	0.75	0.05	100	18.5	1.217	2.149+-0.731	4.589	4.609	5.661+-0.574	6.964
**14**	100	[0.5,0.5]	0.75	0.1	89.5	83.240	1.243	2.693+-0.687	3.807	4.464	6.112 +-0.484	7.196
**15**	100	[0.5,0.5]	0.8	0.05	90.5	94.475	1.365	2.749+-0.628	4.677	3.867	5.999+-0.712	7.108
**16**	100	[0.5,0.5]	0.8	0.1	100	35	1.243	2.273+-0.653	4.834	3.855	5.342+-0.516	6.360
**17**	100	[0.6,0.4]	0.75	0.05	87	100	1.444	3.402+-1.135	6.408	4.018	5.775+-0.655	7.325
**18**	100	[0.6,0.4]	0.75	0.1	99	25.757	1.432	2.437+-0.694	5.056	4.695	6.325+-0.892	7.609
**19**	100	[0.6,0.4]	0.8	0.05	99	16.162	1.713	2.404+-0.309	3.233	4.564	6.021+-0.571	6.963
**20**	100	[0.6,0.4]	0.8	0.1	0.13	92.308	1.561	1.999+-0.332	3.133	5.014	7.267+-0.779	8.036
**21**	100	[0.7,0.4]	0.75	0.05	99.5	31.658	1.243	2.570+-0.785	4.676	4.546	5.524+-0.549	6.616
**22**	100	[0.7,0.4]	0.75	0.1	97.5	87.179	1.359	2.281+-0.591	4.336	3.414	5.834+-0.587	7.433
**23**	100	[0.7,0.4]	0.8	0.05	97.5	100	1.311	2.318+-0.659	4.408	4.019	5.615+-0.521	7.077
**24**	100	[0.7,0.4]	0.8	0.1	98	81.122	1.359	2.165+-0.639	5.394	4.690	6.048+-0.481	7.389
**25**	90	[0.5,0.5]	0.75	0.05	94	96.81	1.244	2.250+-0.788	5.396	3.878	5.518+-0.622	6.971
**26**	90	[0.5,0.5]	0.75	0.1	94	100	1.484	2.668+-0.852	5.320	3.784	5.582+-0.642	7.291
**27**	90	[0.5,0.5]	0.8	0.05	99.5	99.498	1.097	2.251+-0.822	4.955	3.941	5.503+-0.671	7.179
**28**	90	[0.5,0.5]	0.8	0.1	90.5	98.895	1.364	2.244+-0.635	4.743	3.976	5.627+-0.669	7.481
**29**	90	[0.6,0.4]	0.75	0.05	96.5	97.409	1.336	2.368+-0.728	5.220	4.325	5.789+-0.581	7.299
**30**	90	[0.6,0.4]	0.75	0.1	99.5	18.090	1.336	2.514+-0.515	2.502	3.889	5.730+-0.471	6.701
**31**	**90**	**[0.6,0.4]**	**0.8**	**0.05**	**95**	**91.579**	**1.408**	**2.003+-0.381**	**3.419**	**4.119**	**6.052+-0.596**	**7.398**
**32**	90	[0.6,0.4]	0.8	0.1	54.5	99.082	1.364	2.394+-0.676	4.511	4.597	5.756+-0.576	7.372
**33**	90	[0.7,0.4]	0.75	0.05	95	13	1.632	2.281+-0.673	4.041	5.468	7.150+-0.548	7.750
**34**	90	[0.7,0.4]	0.75	0.1	99.5	100	1.336	2.467+-0.700	5.275	4.242	5.684+-0.561	7.525
**35**	90	[0.7,0.4]	0.8	0.05	95.5	98.429	1.254	2.518+-0.797	5.155	4.416	6.060+-0.566	7.902
**36**	90	[0.7,0.4]	0.8	0.1	93.5	100	1.139	2.067+-0.643	5.367	3.762	5.490+-0.603	6.937

Table S3. Results of the grid-search procedure, implemented to identify the best configuration of the self-adaptive deep reinforcement learning optimization strategy for the pIC50 against USP7 and SAS. The best configuration is highlighted in bold. Min – Minimum, Avg – Average +- standard deviation, Max – Maximum

Supplementary Figures

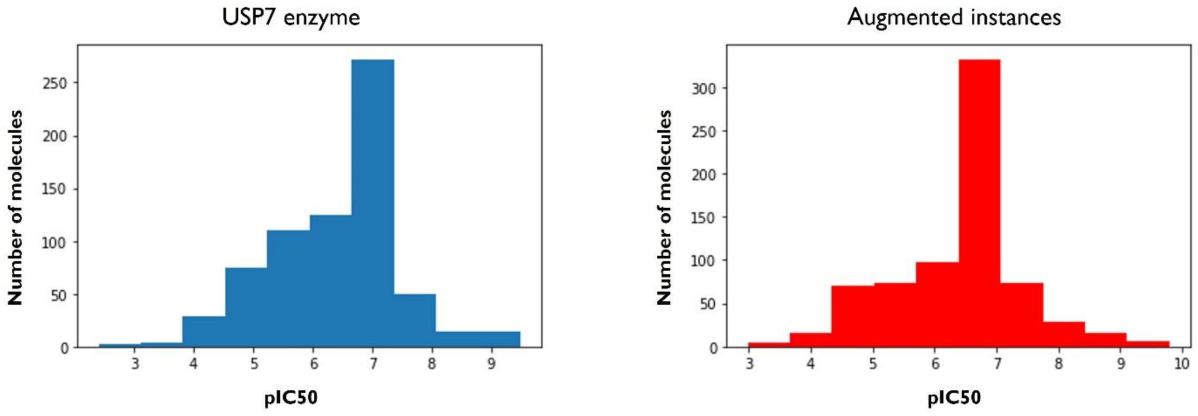

Figure S1. Comparison of the pIC50 distributions: instances extracted from the USP7 target (blue) and molecules obtained to augment the dataset extracted from targets similar to the USP7 (red).

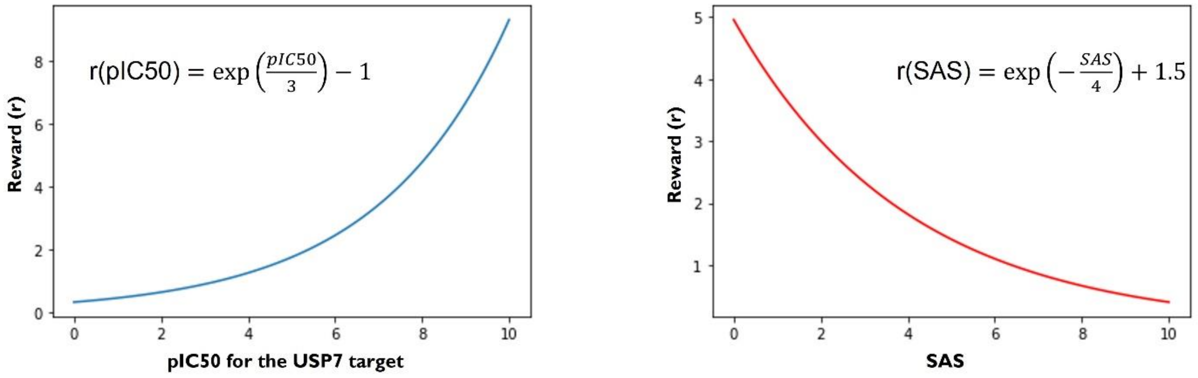

Figure S1. Design of the reward functions for maximizing the pIC50 for the USP7 and minimizing the synthetic accessibility score (SAS).

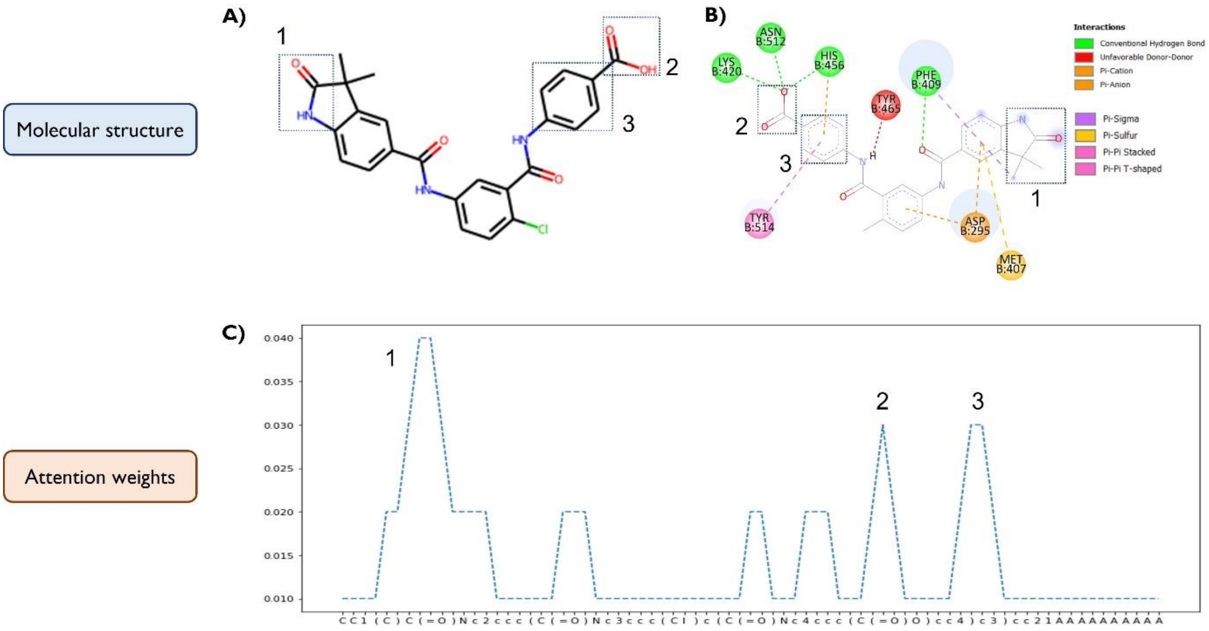

Figure S2. A - Visualization of regions considered as most important for the Predictor. B - Docking simulation results. C - Attention weights across the molecular structure.

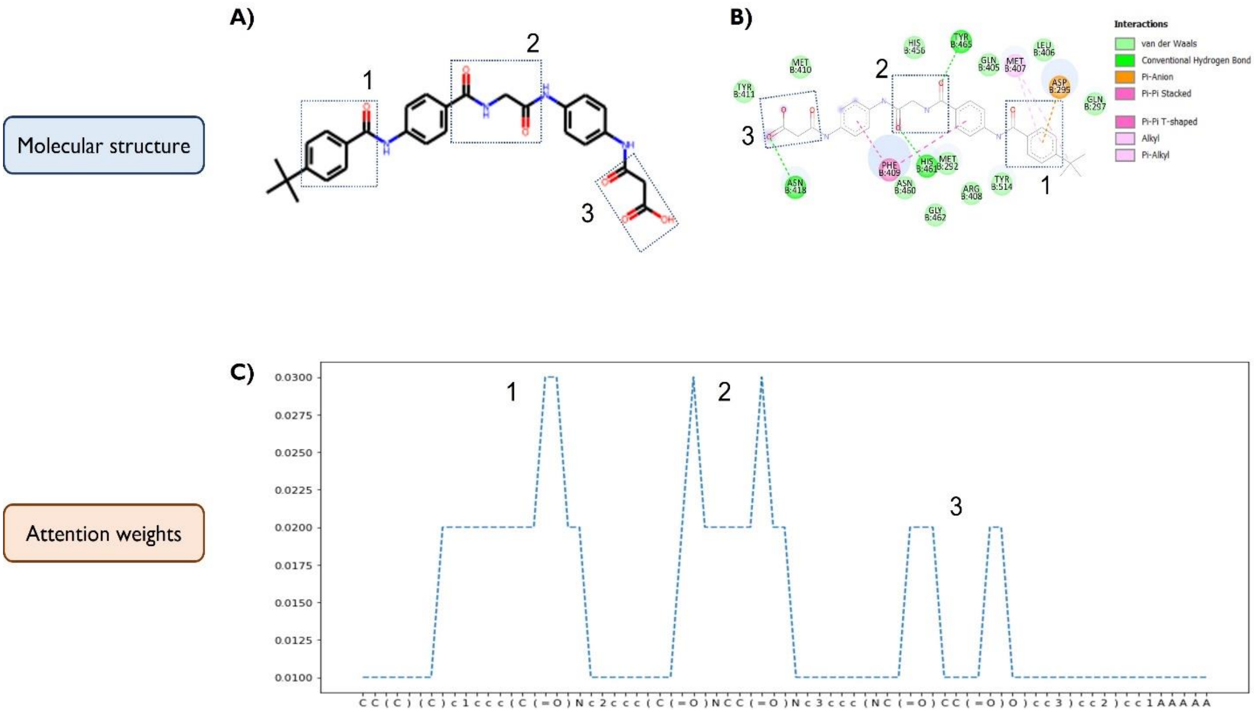

Figure S3. A - Visualization of regions considered as most important for the Predictor. B - Docking simulation results. C - Attention weights across the molecular structure.

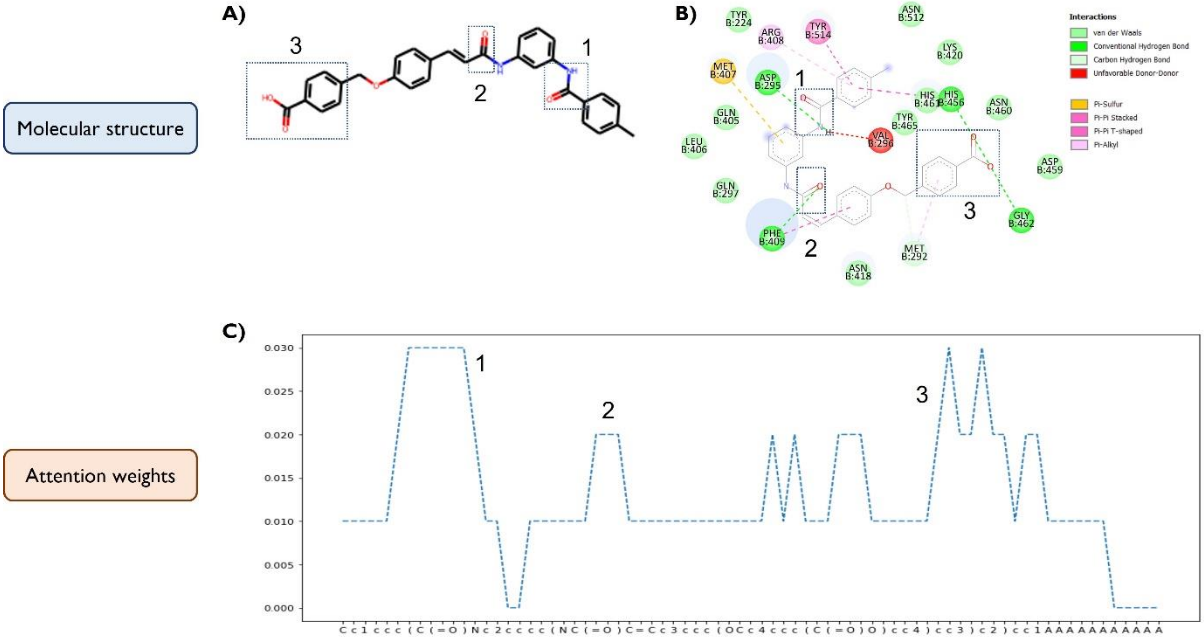

Figure S4. A - Visualization of regions considered as most important for the Predictor. B - Docking simulation results. C - Attention weights across the molecular structure.
